# The Relationship of On-Call Work with Fatigue, Work-Home Interference, and Perceived Performance Difficulties

**DOI:** 10.1155/2015/643413

**Published:** 2015-10-19

**Authors:** Carla M. Ziebertz, Madelon L. M. van Hooff, Debby G. J. Beckers, Wendela E. Hooftman, Michiel A. J. Kompier, Sabine A. E. Geurts

**Affiliations:** ^1^Behavioural Science Institute, Radboud University, Montessorilaan 3, 6525 HR Nijmegen, Netherlands; ^2^Netherlands Organisation for Applied Scientific Research (TNO), Schipholweg 77-89, 2316 ZL Leiden, Netherlands

## Abstract

*Objectives*. This study examined the relationship between on-call duty *exposure* (active and total on-call hours a month, number of calls per duty) and employees' *experiences* of being on-call (stress due to unpredictability, ability to relax during inactive on-call periods, restrictions during on-call duties, on-call work demands, and satisfaction with compensation for on-call duties) on the one hand and fatigue, strain-based and time-based work-home interference (WHI), and perceived on-call performance difficulties (PPD) on the other hand. *Methods*. Cross-sectional survey data were collected among a large heterogeneous sample of Dutch employees (*N* = 5437). The final sample consisted of 157 on-call workers (23–69 years, 71% males). Data were analyzed by means of hierarchical regression analyses (controlling for age and job characteristics). *Results*. Differences in on-call work exposure were not systematically related to fatigue, WHI, and PPD (all *p*'s >0.50). The experience of being on-call explained a medium proportion of the variation in fatigue and strain-based WHI and a medium to large proportion of the variation in time-based WHI and PPD over and above the control variables. *Conclusions*. Our results suggest that it is employees' experience of being on-call, especially the experience of stress due to the unpredictability, rather than the amount of exposure, that is related to fatigue, WHI, and perceived on-call performance difficulties.

## 1. Introduction

On-call work refers to work done on an “as needed basis,” meaning that employees must be available at certain times to be called to work if required by the employer. Typically, this form of scheduling is used to provide 24/7 coverage in facilities where emergencies that need to be dealt with immediately can occur [1]. On-call work occurs in a wide variety of occupations, such as firemen, police officers, doctors, midwives, utility workers, engineers, information technologists, and airline pilots [1, 2].

Previous research has shown that on-call work can have negative effects on employees' well-being and work-related outcomes such as performance and turnover intentions [3–8]. However, most studies have focused on medical staff with on-site standby duties (i.e., duties during which employees remain at the workplace and that count as working time). The aim of the present study was to gain more insight into the consequences of another type of on-call work: “off-site” on-call duties during which employees do not remain at work but can be called to work in case of an emergency. In order to do so, we first examined how exposure to off-site on-call duties relates to fatigue, work-home interference, and performance difficulties. Second, we examined employees' experience of this on-call exposure. Occupational health research has shown that exposure to work can be especially detrimental when employees' experiences of the work are unfavorable (e.g., [9–12]). Therefore, we studied not only “objective” exposure to on-call duties in relation to fatigue, WHI, and performance difficulties, but also how employees' experiences of being on-call relate to these outcome measures.

In the following, the potential consequences of (i) on-call duty exposure and (ii) on-call duty experiences will be discussed.

### 1.1. Exposure to Off-Site On-Call Duties

Exposure to on-call work is likely to affect employees' recovery from work. In The Netherlands and other European countries, off-site on-call duties are officially considered rest time, not working time [13]. Only when an employee is called to work, the active hours are legally considered working hours. This means that employees can be on-call in between regular work-hours, that is, during time that is usually reserved for recovery from work. Recovering from work-related load reactions is critical for employees' well-being and health [14]. According to the effort-recovery model [15], a long-lasting situation of incomplete recovery from load effects (e.g., fatigue) that unavoidably build up due to effort expenditure at work eventually results in chronic load reactions, which, according to allostatic load theory [16], lead to impaired health (e.g., [17, 18]).

There are several ways in which exposure to on-call work can affect recovery. First of all, being called to work during an on-call duty means an interruption of employees' free time, an extension of exposure to work demands, and, thus, less time for recovery. Second, restrictions with regard to location and activities during on-call duties (e.g., having to stay within a certain radius from the workplace and to abstain from alcohol) may interfere with employees' leisure activities and cause work-home interference and thereby impair recovery [6, 10]. Third, it is likely that the restrictions and the possibility of being called to work interfere with the ability to psychologically detach from work. Psychological detachment refers to mentally disengaging from work and not thinking about work-related issues [9]. A lack of detachment relates to negative recovery-related outcomes such as fatigue, work-home interference, and emotional exhaustion (e.g., [19–21]).

In addition to the potential negative effects on employees' well-being, such as fatigue and work-home interference, on-call work might also have negative effects on work performance. Previous research has shown that frequent standby duties are associated with, for example, decreased visual memory, reaction times, vigilance, and clinical performance (e.g., [3, 22–24]). In driving simulation experiments, employees' performance after a standby duty has been found to be comparable to the effect of a 0.05% [23] or even 0.1% blood alcohol concentration [25, 26]. There are several reasons to assume that performance is also reduced during off-site on-call duties. First, during on-call duties, employees can be called to work while not fully recovered from prior work efforts, which may result in a suboptimal condition to perform well. Second, in case of a call, workers are quite abruptly drawn from a nonwork ambiance into a high effort work situation. This “switch” might make it more difficult to optimally direct one's attention towards work-related tasks.

Taken together, there are reasons to believe that exposure to on-call work may have negative consequences such as fatigue, work-home interference, and performance difficulties. Based on the effort-recovery model [15] our first hypothesis is that the amount of exposure to on-call duties is positively related to (a) fatigue, (b) strain-based WHI (occurs when tensions built up at work are transferred to private life [27]), (c) time-based WHI (occurs when work obligations make it timewise impossible to meet obligations in the private domain [27]), and (d) on-call performance difficulties. In the present study, exposure was operationalized as (i) the number of hours a month employees are on call, (ii) the number of active on-call hours (i.e., working hours) a month, and (iii) the average number of calls per on-call duty.

### 1.2. Experience of Being On-Call

As mentioned above, besides factual exposure, we also took employees' experience of the on-call duties into account. Previous research has shown that exposure to work can be especially detrimental when employees' work experiences are unfavorable (e.g., [9–12]). Off-site on-call duties may be experienced unfavorably for several reasons.

First of all, on-call duties can be experienced as stressful due to the high unpredictability and the lack of control over whether or not one will be called to work. Previous research has shown that unpredictability can indeed cause stress [28]. Second, the unpredictability and restrictions may also interfere with employees' ability to relax, which in turn is important for psychological detachment and recovery [9]. Another cause of stress may be that employees are usually only called in case of a critical incident when there is no one else to deal with it. As such, the work may be experienced as taxing and demanding, which in turn may lead to stress [15, 29]. Finally, since on-call duties officially count as rest time, only the actual working hours have to be compensated for [13]. Employees' satisfaction with the compensation they receive is likely to affect the consequences of on-call duties. According to Siegrist's [30] effort-reward imbalance model, perceived imbalance between employees' amount of effort and the rewards they receive for their efforts leads to negative consequences such as fatigue. Furthermore, in case of satisfactory compensation, employees may be more likely to experience their on-call duties as working time, which might make the restrictions more acceptable and on-call duties potentially even desirable.

Based on the effort-recovery model [15], the effort-reward imbalance model [30], and previous research on the relevance of the psychological experience of work (e.g., [6, 10, 11, 31, 32]), our second hypothesis is that the more employees experience their on-call duties as unfavourable, the more (2a) fatigue, (2b) strain-based WHI, (2c) time-based WHI, and (2d) on-call performance difficulties they will show. In the present study, experience of on-call duties was operationalized as (i) the experience of stress due to the lack of control over whether or not being called to work, (ii) the ability to relax during inactive on-call periods, (iii) the perceived on-call work demands, (iv) restrictions, and (v) the satisfaction with the compensation for on-call duties.

## 2. Method

### 2.1. Procedure and Participants

The data were collected by means of an online questionnaire in autumn 2013. The sample was derived from an earlier questionnaire study (Netherlands Working Conditions Survey (NWCS), year 2010) conducted by the Netherlands Organisation for Applied Scientific Research (TNO) and Statistics Netherlands [33]. The link to the questionnaire of the present study was sent to all respondents of the earlier questionnaire who had volunteered to participate in subsequent studies (*N* = 5437). Reminders were sent two and three weeks after the initial invitation. The response rate was 33.1% (*N* = 1798). Out of the 1798 employees, 203 (11.3%) indicated working on-call and were therefore relevant to the present study.

Respondents who indicated working less than 24 hours a week (*n* = 19) were excluded from the present study because work should constitute a substantial part of participant's lives when studying the consequences of work. Furthermore, one participant who indicated working more than 48 hours a week on contract (i.e., more than the legal maximum) was excluded. Likewise, respondents who indicated working less than one or more than 336 hours a month on-call (i.e., more than the legal maximum) were excluded from the present study (*n* = 21). Finally, five respondents were excluded due to missing data on several study variables. This resulted in a final sample of 157 participants (70.7% males).

On average, participants worked 36.2 hours a week on contract (SD = 4.91). Their mean age was 45.0 years (SD = 11.07) with a range from 23 to 69 years. Most of the participants had completed higher vocational education (38.9%) or vocational training (26.1%) and 17.8% had a university degree. About 16.0% of the participants had completed secondary school and 1.2% had completed elementary school or did not complete any education. Seventy-seven percent of the participants were married or cohabiting and 44.6% had children living in their household. Most respondents worked as social workers or in health care professions (22.9%), as specialists (i.e., IT specialists, engineers; 21.7%) or in service professions (14.6%).

### 2.2. Measures

#### 2.2.1. On-Call Duty Exposure

Respondents were asked to indicate the frequency of on-call duties per month, the average duration of those duties in hours, and the time spent working during an average on-call duty. Based on these exposure items, the* number of on-call hours per month* (product of the frequency of on-call duties a month and the duration of one on-call duty) and the* number of active on-call hours per month* (product of the frequency of on-call duties per month and the average amount of time spent working during an average on-call duty) were computed. The average* number of calls per on-call duty* was assessed with the following item: “on average, how many times are you called to work during* one* on-call duty?”

#### 2.2.2. Experience of Being On-Call

Due to a lack of validated scales, all on-call work experiences were assessed with self-developed items. Response scales were based on the well-known Dutch grade notation system ranging from 1 to 10 [34].* Satisfaction with compensation for on-call duties* was assessed with the following item: “how satisfied are you with the compensation of your on-call duties?” (1 = extremely unsatisfied, 10 = extremely satisfied).* On-call work demands* were assessed with the item: “how taxing is the work that you have to do when being called during an on-call duty?” (1 = not at all taxing, 10 = extremely taxing). The experience of restrictions (*on-call restrictions*) was assessed with the item: “to what extent do you feel restricted during on-call duties (e.g., in choosing leisure activities)?” (1 = not at all restricted, 10 = extremely restricted). The ability to relax (*on-call relaxation*) was assessed with the item “how well can you relax during periods in which you do not have to work during on-call duties?” (1 = not at all, 10 = extremely well). Finally, the experience of stress due to unpredictability (*on-call stress*) was assessed with the following: “how stressful do you find not having control over whether or not you will be called during an on-call duty?” (1 = not at all stressful, 10 = extremely stressful).

#### 2.2.3. Fatigue

*Fatigue* was assessed with a shortened version (four items) of the Fatigue Assessment Scale (FAS) [35]. An example item is “I suffer from fatigue.” Answers were provided on a five-point Likert scale (1 = almost never; 2 = sometimes; 3 = regularly; 4 = often; 5 = almost always). Cronbachs' *α* for this scale was 0.84.

#### 2.2.4. Work-Home Interference (WHI)

*Work-home interference (WHI)* was measured with a shortened version of the negative work-home interference scale of the SWING questionnaire [36]. A distinction was made between* strain-based WHI* (3 items; e.g., “how often does it occur that you are irritable at home because your work is demanding?”; *α* = 0.83) and* time-based WHI* (3 items; e.g., “how frequently does it occur that your work schedule makes it difficult for you to fulfill your domestic obligations?”; *α* = 0.78). Responses were provided on a four-point Likert scale (1 = almost never; 2 = sometimes; 3 = often; 4 = almost always).

#### 2.2.5. Perceived On-Call Performance Difficulties (PPD)

PPD were assessed with four items which were based on the Dutch scale for measuring experienced load (Schaal Ervaren Belasting, [29]). An example item is “how much effort does it cost you to complete your tasks without errors when you are called to work during on-call duty?” Respondents had to indicate their answer on a scale from 1 (= no effort at all) to 10 (= extremely much effort). Cronbachs' *α* for this scale was 0.94. It should be noted that fatigue and WHI were assessed as general items (i.e., not in relation to on-call duties), whereas PPD were on-call duty specific.

#### 2.2.6. Control Variables

In order to avoid potential confounding, we included respondents'* gender* (0 = male, 1 = female),* age* (years),* children in the household* (0 = no, 1 = yes), and* cohabiting or marital status* (0 = single, 1 = cohabiting or married) as control variables in the questionnaire. Furthermore, to examine whether the on-call duty variables can explain a significant amount of variance in fatigue, WHI, and PPD over and above general job characteristics, we controlled for three important job characteristics [30].* Job demands* were assessed with four items from the Questionnaire on the Experience and Assessment of Work (VBBA, [37]). An example item is “do you have to work extra hard?” Responses were provided on a four-point Likert scale (1 = almost never; 4 = almost always; *α* = 0.84).* Autonomy* was assessed with three items based on the Job Content Questionnaire [38, 39]. An example item is “can you decide how your work is executed on your own?” Answers were provided on a three-point Likert scale (1 = yes, regularly, 2 = yes, sometimes, and 3 = no). The average score was mirrored for the ease of interpretation, so that higher scores indicate a higher degree of autonomy. Cronbachs' *α* for this scale was 0.80.* Social support* by the supervisor was assessed with three items based on the TNO Work Situation Survey (TAS, [40, 41]). An example item is “my supervisor has an eye for the well-being of his/her employees.” Respondents could indicate the extent to which they agree with the items on a Likert scale from 1 (= totally disagree) to 4 (= totally agree). Cronbachs' *α* for this scale was 0.87.

Finally, in the analyses concerning the experiences of being on-call which included employees' satisfaction with the compensation they receive for their on-call duties, we controlled for* compensation for on-call duties*. This variable included compensation in money, extra free time, or both and was measured with the following item: “do you receive compensation for your on-call duties?” (1=no, 2 = yes, for the actual working hours, and 3 = yes, for the entire on-call duty). Employees who did receive compensation were also asked whether they received compensation in extra free time, money, or both. Preliminary analyses (the results of these analyses can be requested from the first author) revealed no significant differences between the different types of compensation with regard to fatigue, WHI, and PPD. As such, the variable* compensation for on-call duties* was dummy coded (0 = no compensation, 1 = compensation).

## 3. Results

### 3.1. Descriptive Statistics of On-Call Duty Exposure and the Experience of Being On-Call

As can be seen in Table 1, variance in on-call duty exposure was large. On average, employees were 69.31 hours on-call per month (SD = 71.97) with a range from 1 to 336. Half of the respondents spent 48 hours or less on-call per month and only 7.6% were on-call more than 168 hours (i.e., more than one week per month). The mean number of monthly active on-call hours was 13.78 (SD = 22.90) hours with a range from 0 to 180. Nearly one-quarter (24.8%) of the employees indicated that, during an average on-call duty, they are not called to work and 45.9% indicated that, on average, they are called once.

A majority of the participants (57.5%) felt at least somewhat restricted during their on-call duties (score 6 or higher). Nearly one-third (30.6%) of the participants experienced “not having control over whether or not they will be called to work during their on-call duties” as at least somewhat stressful (score 6 or higher) and 26.1% found it at least somewhat difficult to relax during on-call duties (score 5 or lower). More than a third (43.3%) experienced the work they have to do when called as (reasonably) taxing (score 6 or higher). With regard to satisfaction with compensation for on-call work, 45.2% scored 5 or lower, indicating that they were at least somewhat dissatisfied. The descriptive statistics and percentiles of the experience variables are presented in Table 1.

Correlations among all variables under examination are displayed in Table 2. Except for a significant negative correlation between the number of calls per duty and satisfaction with compensation for on-call duties, there were no significant correlations between on-call exposure and the experience of being on-call. Also, the on-call exposure variables showed no significant relationship with any of the “outcome” variables. Experienced on-call work demands showed a significant positive correlation with PPD and time-based WHI; the experience of restrictions showed a significant positive correlation with fatigue and both time- and strain-based WHI. The experience of stress due to the lack of control correlated positively with all dependent variables, and the ability to relax during on-call duties correlated negatively with all four dependent variables. Lastly, satisfaction with compensation was significantly negatively correlated with strain-based WHI and perceived performance difficulties. Preliminary multivariate analyses revealed that the control variables gender, marital status, and children in the household were not related to the outcome variables (all *p* > 0.05); therefore these variables were excluded from further analyses. The descriptive statistics of the included control variables can be found in Table 1.

### 3.2. On-Call Work Exposure in relation to Fatigue, WHI, and PPD

In order to test the first hypothesis, four two-step hierarchical multiple regression analyses were conducted (i.e., one for each of the dependent variables: fatigue, strain-based WHI, time-based WHI, and PPD). The control variables (i.e., age, job demands, autonomy, and social support) were entered in step one of the regression and the exposure variables (number of hours on-call per month, number of active on-call hours per month, and average number of calls per duty) were entered as predictors in step two.

The results showed that the number of hours on-call per month, the number of active on-call hours per month, and average number of calls per duty did not explain an additional, significant proportion of variance in either fatigue (Δ*R* ^2^ = 0.01, *F*(3,145) = 0.38, and *p* = 0.770), perceived on-call performance difficulties (Δ*R* ^2^ = 0.00, *F*(3,145) = 0.15, and *p* = 0.931), strain-based WHI (Δ*R* ^2^ = 0.01, *F*(3,145) = 0.32, and *p* = 0.813), or time-based WHI (Δ*R* ^2^ = 0.01, *F*(3,145) = 0.68, and *p* = 0.567) over and above the control variables. As such, Hypothesis 1 was not supported.

### 3.3. Experience of Being On-Call in relation to Fatigue, WHI, and PPD

Hypothesis 2 was tested with four two-step hierarchical multiple regression analyses. The four dependent variables were fatigue, strain-based WHI, time-based WHI, and PPD. The control variables (i.e., age, compensation for on-call duties, job demands, autonomy, and social support) were entered in step one of the regression; the experience variables (i.e., on-call stress due to unpredictability, on-call relaxation, on-call restrictions, on-call work demands, and satisfaction with compensation for on-call duties) were entered as predictors in step two.

#### 3.3.1. Fatigue

The first analysis revealed that the control variables (model 1) explained 11.4% of the variation in fatigue and that the experience of being on-call (model 2) explained an additional 6.8%. This change in *R* ^2^ was significant (*F*(5,142) = 2.36, *p* = 0.043). As can be seen in Table 3, the level of stress experienced during on-call duties due to the lack of control was positively related to fatigue. Contrary to our expectations, the level of experienced work demands was negatively related to fatigue, and the experience of restrictions, the ability to relax during on-call duties, and the satisfaction with compensation for on-call duties were not significantly related to fatigue. As such, Hypothesis (2a) was only partly supported.

#### 3.3.2. Work-Home Interference

With regard to strain-based WHI, the control variables (model 1) explained 12.4% of the variation and the experience of being on-call (model 2) explained an additional 9.0%. This change in *R* ^2^ was significant (*F*(5,142) = 3.24, *p* = 0.008). As can be seen in Table 4, stress experienced during on-call duties due to the lack of control was positively related to strain-based WHI. Contrary to our expectations, satisfaction with compensation for on-call duties, on-call restrictions, and on-call relaxation were not significantly related to this outcome measure, and the level of on-call work demands was negatively related to strain-based WHI. Consequently, Hypothesis (2b) was only partly confirmed.

With regard to time-based WHI, the control variables (model 1) explained 19.7% of the variation, and the experience of being on-call (model 2) explained an additional 12.2%. This change in *R* ^2^ was significant (*F*(5,142) = 5.07, *p* < 0.001). As can be seen in Table 5, only the experiences of stress and restrictions during on-call duties were significantly positively related to time-based WHI. The experienced level of on-call work demand, the ability to relax during on-call duties, and the satisfaction with compensation for on-call duties were not significantly related to time-based WHI. As such, Hypothesis (2c) was partly confirmed as well.

#### 3.3.3. Perceived On-Call Performance Difficulties

The control variables (model 1) accounted for 9.4% of the variation in PPD; the experience of being on-call (model 2) accounted for an additional 12.0% of the variation. This change in *R* ^2^ was significant (*F*(5,142) = 4.34, *p* = 0.001). As can be seen in Table 6, only the level of on-call work demands was positively related to PPD. Satisfaction with compensation for on-call duties showed a marginal negative relationship with PPD. The experience of restriction, the experience of stress, and the ability to relax during on-call work were not significantly related to PPD. As such, Hypothesis (2d) was partly supported.

## 4. Discussion

### 4.1. Discussion of the Results

Off-site on-call duties are an interesting yet relatively understudied working time arrangement [1, 6]. Therefore, we aimed to gain more insight into the relationship between the exposure to off-site on-call duties and the experience of being on-call on the one hand and fatigue, strain-based and time-based WHI, and PPD on the other hand.

In the present study sample, there was a large variation in the amount of exposure to on-call work. Contrary to our first hypothesis, differences in exposure to on-call work were not systematically related to any of the outcome variables. This is not in line with previous research conducted among physicians that found frequent on-call duties to be related to distress and turnover intentions [7]. However, physicians' on-call duties mostly take place during the night, and night shifts have been shown to be negatively related to employees' well-being and health, possibly due to sleep deprivation [42]. This might explain why the frequency of on-call duties has been found to have negative consequences in previous research but not in the present study, where 93% of the employees indicated that their on-call duties took place during the day as well. Another explanation for the nonsignificant results may lie in our exposure measures. Due to the heterogeneity of our study sample, we did not have access to individual on-call schedules and we tried to get insight into the participants' average exposure to on-call duties by means of multiple items on average exposure. However, when schedules vary a lot, it may not be easy for each employee to indicate the average number of on-call duties a month. Also, the average time spent working during one duty and the average number of calls may show large intraindividual variation. In order to be able to draw clear conclusions about the relationship between off-site on-call duty exposure, fatigue, WHI, and PPD, further research with larger samples that include different professions and different types of on-call duties (e.g., with regard to the length and timing) is needed. In addition to self-reports, company registered data of on-call work exposure should be used. Furthermore, a repeated measurements diary study in which participants keep track of their on-call hours and momentary experiences might provide more insight into the relationship between the actual on-call duty exposure and fatigue, work-home interference, and performance. Multiwave diary designs would also allow disentangling the effects of different types of on-call shifts (e.g., night shifts and day shifts).

The second hypothesis was partly confirmed. Many employees experienced their on-call duties as (somewhat) unfavourable. The experience of being on-call in turn was related to fatigue, strain-based and time-based WHI, and on-call performance difficulties, but not all experiences contributed significantly to the prediction. All in all, on-call stress (i.e., the experience of stress due to the unpredictability of being on-call) seemed to be the most important predictor as it was positively related to all “outcome” variables except for performance difficulties. Employees' satisfaction with the compensation they received for their on-call duties and their ability to relax during inactive on-call work periods were not related to either fatigue, WHI, or PPD when controlling for important job characteristics. Feeling restricted during on-call work was related to the most proximal criterion, that is, time-based WHI, but did not explain any additional variance in strain-based WHI, PPD, or general fatigue over and above the control variables. Contrary to what we expected based on the effort-recovery model [15], on-call work demands were negatively related to fatigue and strain-based WHI. This result is theoretically implausible. In post hoc analyses (the results of these analyses can be requested from the first author) with (i) fatigue and (ii) strain-based WHI as dependent variables, we compared what happens when on-call work demand is the only predictor besides the control variables to what happens when on-call work stress is the only predictor. For both dependent variables, the effect sizes of on-call demands and on-call stress are similar, but whereas the significant effects of on-call demands only appear when this variable is entered into the analysis in combination with the other experiences, this is not the case for on-call stress (which is a significant predictor when entered alone as well as with the other predictors). In other words, the effects of on-call stress remain significant when it is the only predictor besides the control variables. Furthermore, the Pearson correlations between on-call work demands and both dependent variables were not significant. Hence, we interpret the significant results for on-call demands as an artefact. Further research is needed to examine the role of on-call work demands in more detail.

### 4.2. Strengths and Limitations

The limited previous studies on on-call duties focused on only one profession (mostly medical staff), thereby limiting the external validity of the results [1]. Therefore, one asset of the present study is the sample that consisted of a heterogeneous group of employees with different professions and from different organizations. Also, previous studies have mainly focused on on-site standby duties, so another asset is the focus on off-site on-call duties which have been largely neglected so far. Furthermore, to the authors' knowledge, the present study was the first to investigate both exposure to on-call work duties and their psychological significance (in terms of experiences) in relation to general “outcomes” such as fatigue and work-home interference and an on-call duty specific performance indicator, that is, perceived difficulties to perform well when called to work. Moreover, our analyses were quite strict. We controlled for important general job characteristics, thereby minimizing the possibility of confounding effects.

Nonetheless, several limitations of the present study warrant further research. First, the validity of the on-call duty exposure items is not without problems. Individual scores were highly variable and not always plausible (e.g., a small percentage reported considerably more on-call hours than the legal maximum or more active on-call hours than total on-call hours a month). Respondents producing such errors were excluded from the analyses, but it is possible that our self-developed items were not clear to some participants. As mentioned above, less valid measures might be a reason for the null-findings on on-call exposure. Future research among heterogeneous samples may benefit from more valid measures of on-call duty exposure (e.g., official work schedules).

Second, the present study was cross-sectional, so no causal relations can be implied. With regard to employees' experience of being on-call, bidirectionality of effects might be plausible. For instance, general fatigue and work-home interference may affect employees' experience of being on-call. Further research with longitudinal or experimental designs is needed to investigate the causal direction of the associations.

### 4.3. Implications

Since on-call duties are officially considered rest time and take place during time meant for recovery [13], the results of the present study are reason for some concern. About one-third of the employees experienced their on-call duties as (reasonably) stressful, which may impair their recovery. Recovery, however, is critical for employees' well-being and health [15, 16]. Working time legislation only takes the length and frequency of on-call duties into account, but the results of the present study suggest that the psychological aspect (i.e., employees' experience) of on-call duties may be more important than the number of hours spent on-call and that even short or infrequent on-call periods may interfere with employees' well-being, thereby presenting a risk for ill health. Employers should therefore pay attention to how employees experience their on-call duties and lighten the on-call burden of employees prone to suffer from stress in order to prevent negative consequences such as fatigue and WHI, which may, in the long run, lead to health problems.

## 5. Conclusions

In sum, the present study showed that employees' experience of their on-call duties is related to their general fatigue, work-home interference, and the difficulties they have in performing well when called to work, even when controlling for important job characteristics. Our results suggest that it is the experience of being on-call rather than the variation in exposure to on-call duties itself that is associated with negative outcomes. This means that even a low amount of (active) on-call hours a month and even a low frequency of being called to work can be related to an increase in fatigue and work-home interference, when employees experience being on-call negatively. Therefore, employees' experience of on-call duties should be included in future studies on on-call work. In addition, future research should be conducted to gain more insight into potential predictors of the experience of being on-call (e.g., individual characteristics) and to investigate how the experience of being on-call can be improved, in order to form a basis from which to develop successful interventions that decrease the negative consequences of being on-call.

## Figures and Tables

**Table 1:** Descriptive statistics of the main variables (*n* = 157).

	Range	Min	Max	Mean	SD	Percentiles		
						25%	50%	75%
On-call duty exposure								
Number of hours on-call per month		1.00	336.00	69.31	71.97	12.00	48.00	100.00
Number of active on-call hours per month		0.00	180.00	13.78	22.90	1.00	6.00	16.00
Average number of calls per duty		0.00	30.00	1.74	3.49	0.50	1.00	2.00

Experience of being on-call								
On-call relaxation	1–10	1.00	10.00	6.94	2.46	5.00	7.00	9.00
On-call stress	1–10	1.00	10.00	3.95	2.57	1.50	3.00	6.00
On-call work demands	1–10	1.00	10.00	4.78	2.60	3.00	6.00	8.00
On-call restrictions	1–10	1.00	10.00	5.86	2.78	2.00	5.00	7.00
Satisfaction with compensation for on-call work	1–10	1.00	10.00	5.76	2.83	3.50	6.00	8.00

Strain-based WHI	1–4	1.00	3.67	1.74	0.61			
Time-based WHI	1–4	1.00	3.67	1.75	0.59			
Fatigue	1–5	1.00	5.00	2.15	0.82			
Perceived on-call performance difficulties	1–10	1.00	8.00	2.43	1.66			

Control variables								
Age		23.00	69.00	45.01	11.07			
Compensation^a^	0-1	0.00	1.00	0.77	0.42			
Job demands	1–4	1.25	4.00	2.57	0.58			
Autonomy	1–3	1.00	3.00	2.46	0.59			
Social support	1–4	1.00	4.00	3.15	0.64			

^a^0 = no compensation, 1 = compensation.

**Table 2:** Correlations among the variables under examination (*n* = 157).

	1	2	3	4	5	6	7	8	9	10	11	12	13	14	15	16	17	18	19
(1) # of on-call hours per month																			
(2) # of active on-call hours per month	.33^*∗∗*^																		
(3) # of calls per duty	.21^*∗∗*^	.21^*∗∗*^																	
(4) On-call relaxation	.01	.05	−.06																
(5) On-call stress	−.00	.05	.13	−.46^*∗∗*^															
(6) On-call work demands	.06	.07	.13	−.17^*∗*^	.43^*∗∗*^														
(7) On-call restrictions	.07	−.01	.16	−.29^*∗∗*^	.50^*∗∗*^	.27^*∗∗*^													
(8) Satisfaction with compensation	−.13	−.05	−.18^*∗*^	.16^*∗*^	−.23^*∗∗*^	−.18^*∗*^	−.21^*∗∗*^												
(9) Fatigue	−.09	−.10	.05	−.21^*∗∗*^	.26^*∗∗*^	.01	.24^*∗∗*^	−.09											
(10) Strain-based WHI	−.09	−.04	.08	−.17^*∗*^	.29^*∗∗*^	.02	.24^*∗∗*^	−.11	.69^*∗∗*^										
(11) Time-based WHI	−.04	.03	.15	−.28^*∗∗*^	.41^*∗∗*^	.16^*∗*^	.37^*∗∗*^	−.22^*∗∗*^	.41^*∗∗*^	.51^*∗∗*^									
(12) PPD	−.02	.01	.01	−.22^*∗∗*^	.30^*∗∗*^	.33^*∗∗*^	.10	−.25^*∗∗*^	.24^*∗∗*^	.25^*∗∗*^	.30^*∗∗*^								
(13) Age	−.07	.09	−.05	.17^*∗*^	−.15	−.05	−.15	.06	−.10	−.03	−.16^*∗*^	−.11							
(14) Gender^a^	−.05	−.18^*∗*^	.08	−.13	.10	.03	.20^*∗*^	.02	.05	−.08	−.02	−.12	−.21^*∗∗*^						
(15) Marital status^b^	−.01	−.04	.01	−.09	−.01	.05	−.02	−.02	−.05	.02	−.01	−.03	−.05	.09					
(16) Children in household^c^	.02	.07	.03	−.05	.00	.04	.11	.14	.06	.06	.10	.02	−.03	−.04	.29^*∗∗*^				
(17) Compensation^d^	.09	−.1	.12	−.11	−.01	.08	.11	.14	.14	−.05	−.11	−.05	.02	−.02	.09	.07			
(18) Job demands	−.07	−.12	.17^*∗*^	−.21^*∗∗*^	.21^*∗∗*^	.18^*∗*^	.10	−.05	.21^*∗∗*^	.30^*∗∗*^	.33^*∗∗*^	.21^*∗∗*^	.05	.09	−.00	−.02	.00		
(19) Autonomy	.10	−.07	−.03	.22^*∗∗*^	−.27^*∗∗*^	−.12	−.23^*∗∗*^	.26^*∗∗*^	−.13	−.04	−.25^*∗∗*^	−.15	.23^*∗∗*^	−.25^*∗∗*^	.11	.08	.04	−.13	
(20) Social Support	.15	.00	.03	.11	−.16	−.24^*∗∗*^	−.24^*∗∗*^	.22^*∗∗*^	−.22^*∗∗*^	−.22^*∗∗*^	−.22^*∗∗*^	−.21^*∗*^	−.01	−.01	.14	.03	.04	−.20^*∗*^	.30^*∗∗*^

^*∗*^ *p* < .05. ^*∗∗*^ *p* < .01.

^a^0 = male, 1 = female.

^b^0 = single, 1 = cohabiting or married.

^c^0 = no children living in the household, 1 = child(ren) living in the household.

^d^0 = no compensation for on-call duties, 1 = compensation for on-call duties.

**Table 3:** Summary of hierarchical regression predicting fatigue from the experience of being on-call.

Variable	Model 1			Model 2		
	*B*	SE *B*	*β*	*B*	SE *B*	*β*
Age	−.01	.01	−.11	−.01	.01	−.08
Compensation for on-call duties	.30	.15	.15	.30	.15	.16^*∗*^
Job demands	.25	.11	.18^*∗*^	.22	.11	.16^*∗*^
Autonomy	.02	.12	.02	.02	.12	.02
Social support	−.24	.11	−.18^*∗*^	−.24	.11	−.18^*∗*^
On-call relaxation				−.01	.03	−.04
On-call stress				.07	.03	.21^*∗*^
On-call work demands				−.06	.03	−.20^*∗*^
On-call restrictions				.02	.03	.09
Satisfaction with compensation for on-call duties				−.01	.02	−.03

*R* ^2^	.114			.182		
Δ*R* ^2^	.114			.068		
*F* for change in *R* ^2^	3.797^*∗∗*^			2.361^*∗*^		

^*∗*^ *p* < .05. ^*∗∗*^ *p* < .01.

**Table 4:** Summary of hierarchical regression predicting strain-based WHI from the experience of being on-call.

Variable	Model 1			Model 2		
	*B*	SE *B*	*β*	*B*	SE *B*	*β*
Age	−.00	.00	−.06	.00	.00	−.02
Compensation for on-call duties	−.06	.11	−.04	−.05	.11	−.04
Job demands	.29	.08	.28^*∗∗*^	.27	.08	.25^*∗*^
Autonomy	.06	.09	.06	.13	.09	.13
Social support	−.18	.08	−.19^*∗*^	−.17	.08	−.18^*∗*^
On-call relaxation				.00	.02	−.01
On-call stress				.06	.02	.25^*∗*^
On-call work demands				−.05	.02	−.20^*∗*^
On-call restrictions				.03	.02	.12
Satisfaction with compensation for on-call duties				−.01	.02	−.03

*R* ^2^	.124			.214		
Δ*R* ^2^	.124			.090		
*F* for change in *R* ^2^	4.159^*∗∗*^			3.241^*∗∗∗*^		

^*∗*^ *p* < .05. ^*∗∗*^ *p* < .01. ^*∗∗∗*^ *p* < .001.

**Table 5:** Summary of hierarchical regression predicting time-based WHI from the experience of being on-call.

Variable	Model 1			Model 2		
	*B*	SE *B*	*β*	*B*	SE *B*	*β*
Age	−.01	.00	−.15	−.01	.00	−.09
Compensation for on-call duties	−.13	.10	−.10	−.16	.10	−.11
Job demands	.31	.08	.30^*∗∗∗*^	.26	.07	.25^*∗∗*^
Autonomy	−.14	.08	−.14	−.05	.08	−.05
Social support	−.12	.07	−.13	−.07	.07	−.07
On-call relaxation				−.01	.02	−.05
On-call stress				.05	.02	.20^*∗*^
On-call work demands				−.01	.02	−.06
On-call restrictions				.04	.02	.20^*∗*^
Satisfaction with compensation for on-call duties				−.02	.02	−.07

*R* ^2^	.197			.319		
Δ*R* ^2^	.197			.122		
*F* for change in *R* ^2^	7.213^*∗∗∗*^			5.070^*∗∗∗*^		

^*∗*^ *p* < .05. ^*∗∗*^ *p* < .01. ^*∗∗∗*^ *p* < .001.

**Table 6:** Summary of hierarchical regression predicting PPD from the experience of being on-call.

Variable	Model 1			Model 2		
	*B*	SE *B*	*β*	*B*	SE *B*	*β*
Age	−.02	.01	−.10	−.01	.01	−.08
Compensation for on-call duties	−.16	.31	−.04	−.14	.30	−.03
Job demands	.52	.23	.18^*∗*^	.33	.23	.12
Autonomy	−.16	.24	−.06	.02	.23	.01
Social support	−.43	.22	−.16^*∗*^	−.28	.21	−.11
On-call relaxation				−.06	.06	−.08
On-call stress				.09	.07	.14
On-call work demands				.14	.05	.22^*∗*^
On-call restrictions				−.08	.05	−.13
Satisfaction with compensation for on-call duties				−.09	.05	−.16

*R* ^2^	.094			.214		
Δ*R* ^2^	.094			.120		
*F* for change in *R* ^2^	3.034^*∗*^			4.344^*∗∗*^		

^*∗*^ *p* < .05. ^*∗∗*^ *p* < .001.
